# Repair of Lumbosacral Meningomyeloceles With Acelluar Cadaveric Dermal Matrix: An Added Layer of Protection

**Published:** 2008-01-25

**Authors:** Richard L. Agag, Mark S. Granick, Parham Ganchi, Ramazi Datiashvilli, Jeffrey Catrambone

**Affiliations:** Department of Surgery, Division of Plastic Surgery, New Jersey Medical School, University of Medicine and Dentistry of New Jersey, Newark, NJ; Department of Neurosurgery, New Jersey Medical School, University of Medicine and Dentistry of New Jersey, Newark, NJ

## Abstract

**Objective:** Providing adequate soft tissue cover while preventing wound breakdown and infection can present a challenge when repairing large meningomyeloceles. Adding an extra barrier to protect the underlying dural elements in the event of complications should lower the morbidity and mortality associated with large repairs, which are at risk of dehiscence and subsequent exposure of the neural elements. **Methods:** Acellular cadaveric dermal matrix (ACDM) (AlloDerm, Life Cell Corporation, Branchburg, New Jersey) in freeze-dried sheets (thin, 0.2 mm and 0.4 mm), fixed with chromic sutures and placed over the dural repair and underneath associated soft tissue coverage/skin, which in our cases included lumbar fascial flaps, latissimus dorsi flaps, and skin flaps. The neural tube defects were repaired by neurosurgery, and plastic surgery performed the surface closure. A layer of ACDM was placed over the dural repair, fixed in place with chromic suture, and then covered with skin and soft tissue flaps. **Results:** In the series of 12 patients, there were 2 cases of wound dehiscence, one of which required secondary repair and closure. There were no long-term sequelae in our series. **Conclusion:** ACDM can be used as an added layer of protection in neurosurgical repair of large meningomyeloceles that are at risk for dehiscence.

The repair of lumbosacral meningomyeloceles involves closure of exposed neural elements, which are then covered with vascularized tissue. For smaller defects, the repair is usually uneventful and can be done primarily with minimal risk of postoperative wound complications. However, for larger defects (>5 cm diameter), potential complications arise including infection, wound dehiscence, and flap failure. Such adverse events can subsequently complicate the neurosurgical repair. The placement of acellular cadaveric dermal matrix (ACDM) over the neurological repair provides an additional safety layer in preventing dural exposure. We have previously published an article presenting our experience with ACDM in neurosurgical reconstruction. 1 In this article, we present our continued experience using ACDM for closure of large meningomyeloceles. Because neural tube anomalies are a common congenital anomaly, it is important to improve upon the procedure when repairing these larger defects.

## METHODS

A retrospective analysis was performed at the University Hospital, Newark, New Jersey. All 12 patients operated on by pediatric neurosurgery and plastic surgery for closure of large lumbosacral meningomyelocele (>5 cm in diameter), from January 2002 to July 2007, were included. In each patient, ACDM was used to cover the dural repair. Data were gathered from hospital medical records and physician office charts. Data included age, size of defect, intervention, complications, and surgical outcome.

## RESULTS

Twelve patients were identified. One patient was 7 years old, in all others the initial surgery was performed in the first 48 hours of life. In all 12 patients, ACDM was placed over the dural repair. There were a total of 2 wound-healing complications, which all ultimately healed secondarily. One patient was reoperated on and additional ACDM was added to cover dural exposures. No other immediate complications were noted. There have been no long-term complications with the ACDM or neurological sequelae reported with all 12 patients in the 5 years since ACDM was initially used for meningomyelocele repair.

## CASE REPORT 1

**Figure 1:**
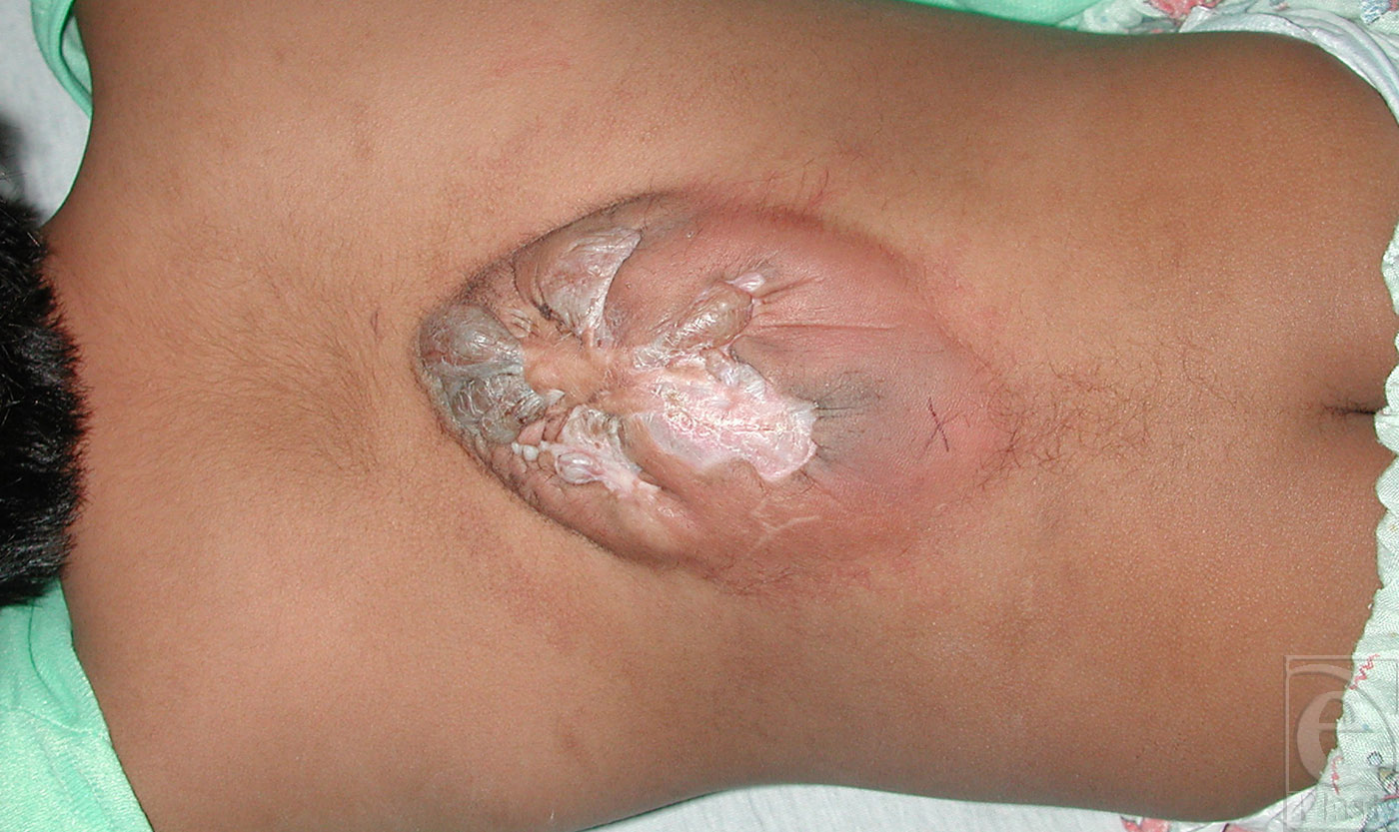
Extensive wound of seven-old with untreated spina bifida.

**Figure 2:**
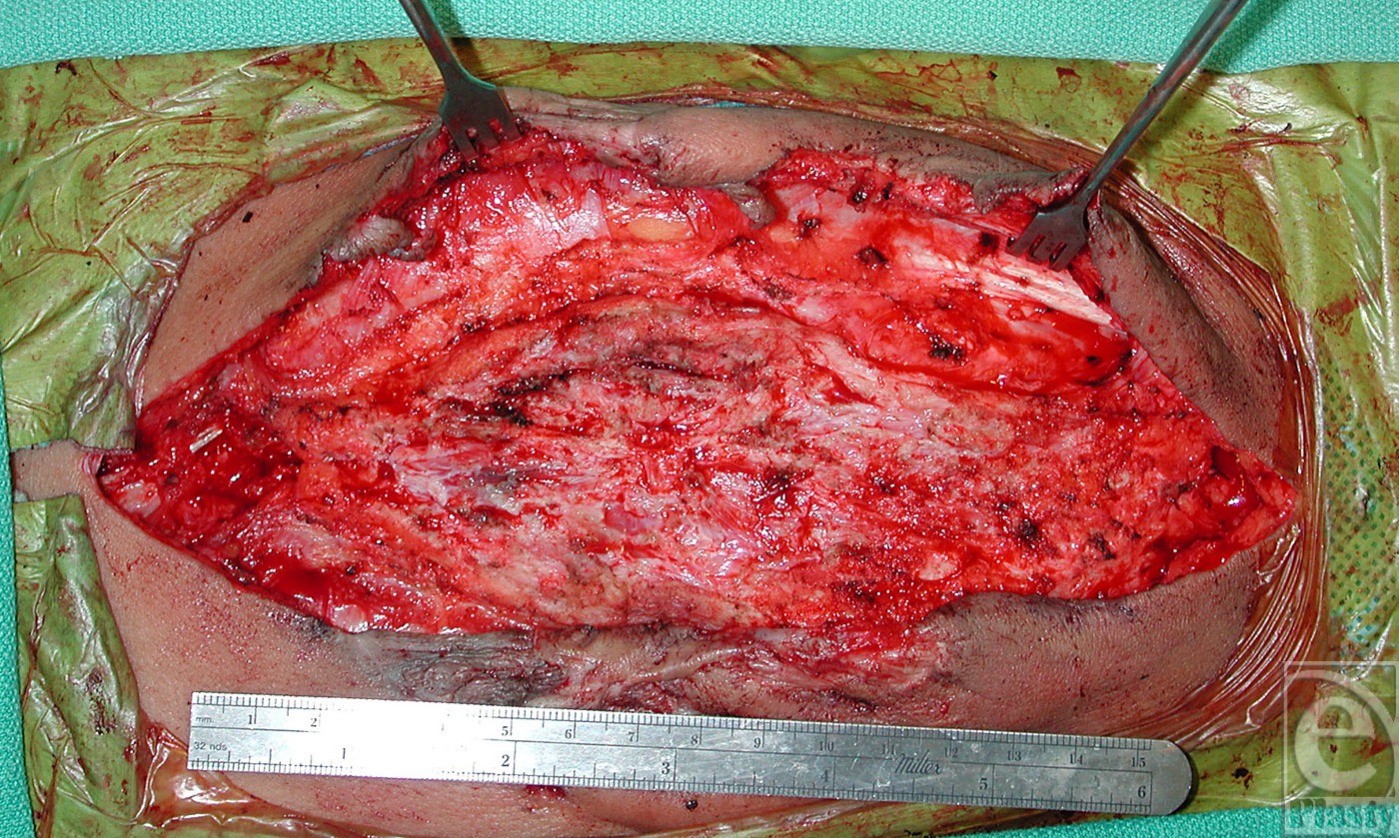
Elevation of latissimus flaps.

**Figure 3:**
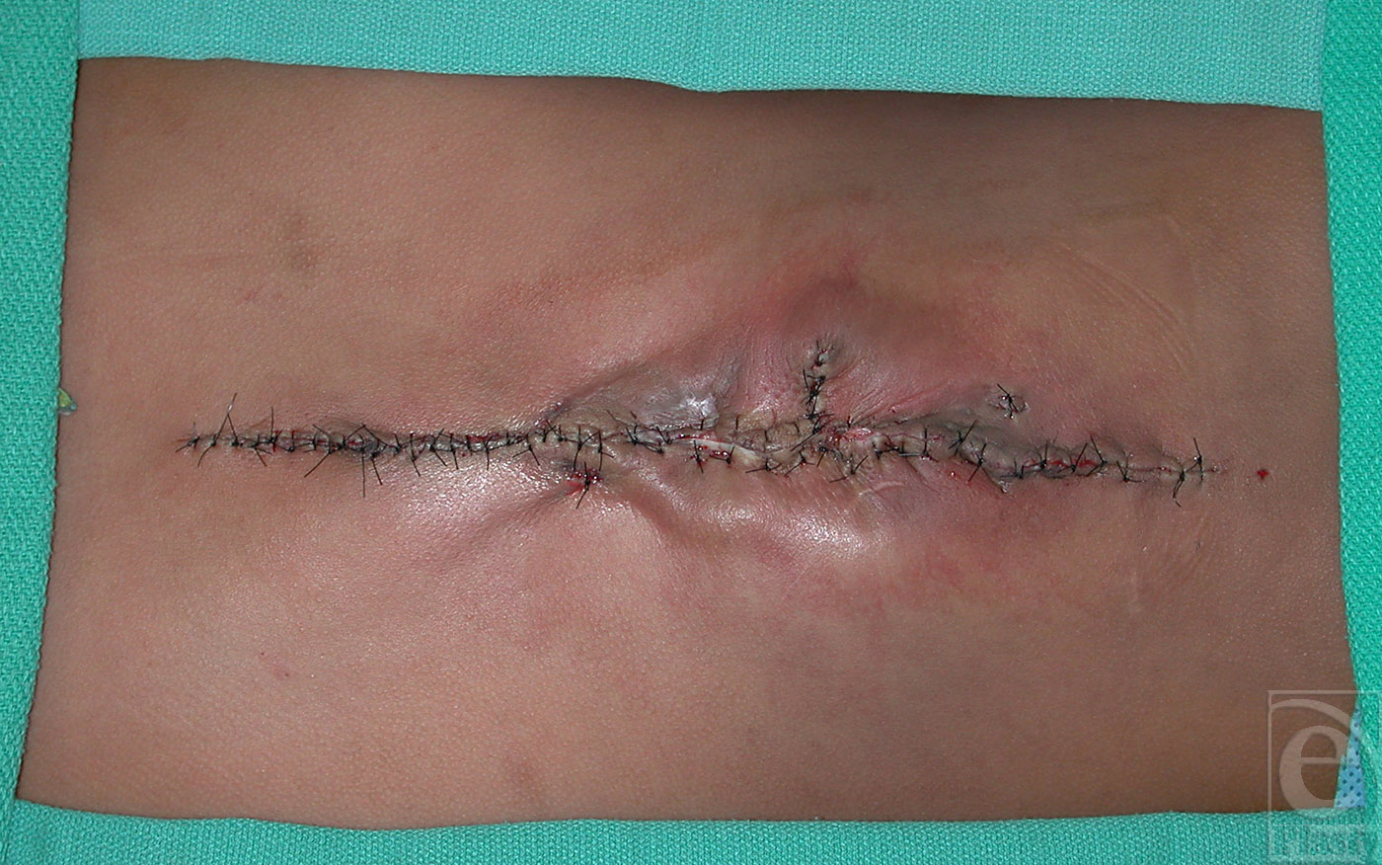
Midline closure with ACDM covering dural repair.

**Figure 4:**
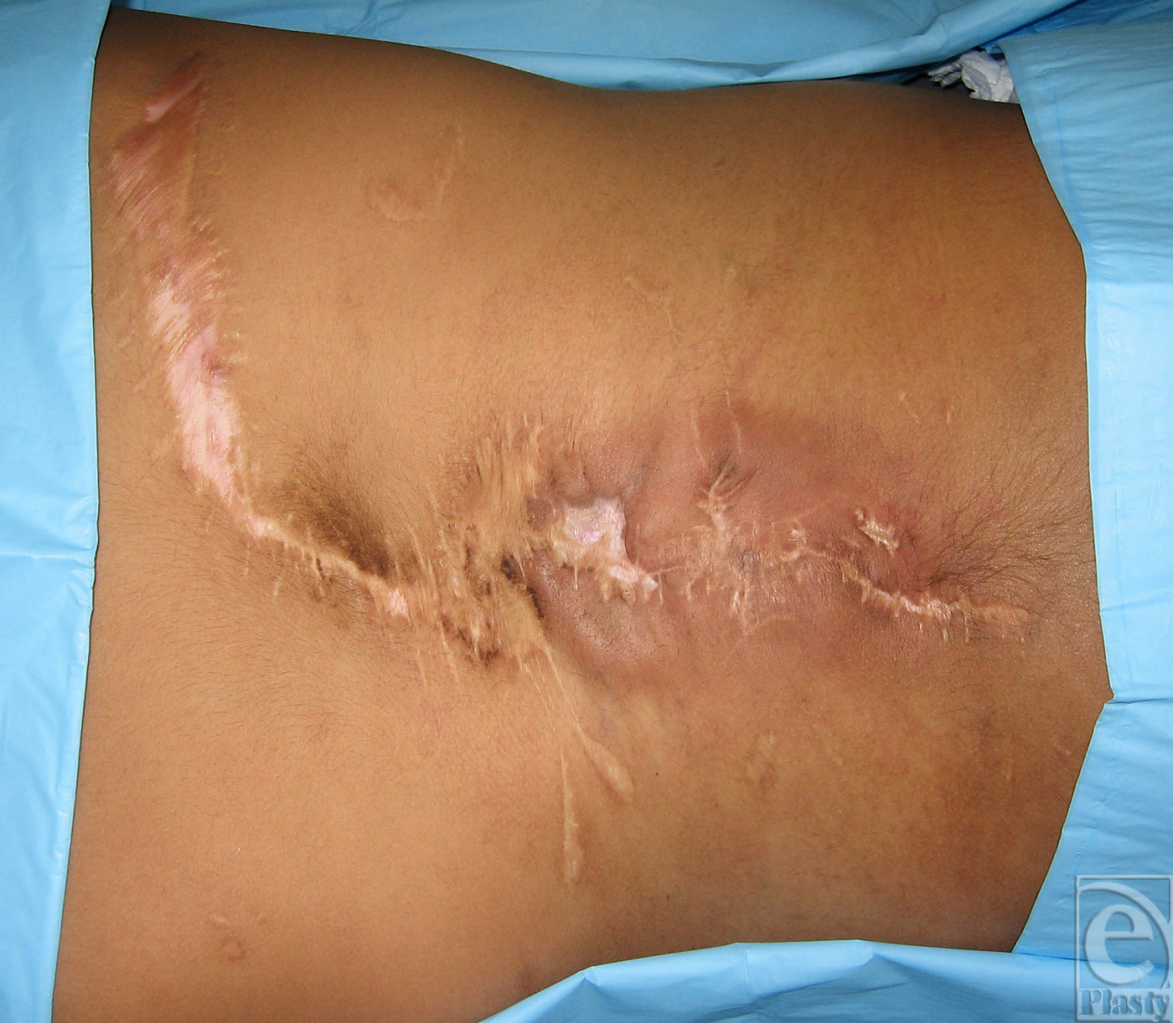
Two year post-operative.

This 7-year-old boy from South America presented with previously untreated spina bifida. At the time of surgery, the neurosurgeon attempted to correct the defect but was unable to close the dura over the spinal cord. Plastic surgery was asked to provide healthy tissue for coverage of the area as well as the spinal elements. The wound extended from the scapula to the iliac crest (15 cm × 8 cm) with the widest portion in the middle of the wound (Fig 1). The involved skin was thin, scarred, and generally of poor quality. Latissimus dorsi flaps were raised laterally to the midaxillary line (Fig 2). The area was sprayed with fibrin glue (Tisseal, Baxter, Deerfield, Illinois) and a piece of ACDM was placed over the central portion of the defect where the spinal elements were exposed. A large midline closure was performed (Fig 3). The patient developed a fluid collection and a small dehiscence in the upper part of the flap. The wound was reexplored and the internal repair had dehisced. Additional ACDM was now placed over the upper part of the defect, as it had not been in the first operation. The flaps were re-elevated and closed in layers with retention sutures. Small areas of wound dehiscence recurred and were allowed to heal secondarily now because ACDM was in place over the extent of the underlying spinal closure. The patient did not have any neurological complications or adverse reactions to the ACDM at 2-year follow-up (Fig 4).

## CASE REPORT 2

The patient was born with a meningomyelocele after elective cesarean section at 27 weeks of gestation. Neurosurgery was able to reduce the placode and repair the dura; however, because of the large size of the defect and the small size of the neonate, plastic surgery was consulted for skin closure. After meningomyelocele repair, the patient was left with a 9 cm × 5 cm defect in the midline. The skin around the incision was thin but appeared viable. Lumbar fascial flaps were raised and then rotated to the midline. A 4 cm × 2 cm piece of ACDM was placed and anchored in place with 5–0 chromic catgut. The skin was then closed above this. Three days after the operation, a 1 cm × 2 cm area of skin dehisced in the middle of the wound exposing the ACDM but not the dura. Because the ACDM was in place, the wound was allowed to heal in secondarily without complication.

## CASE REPORT 3

**Figure 5:**
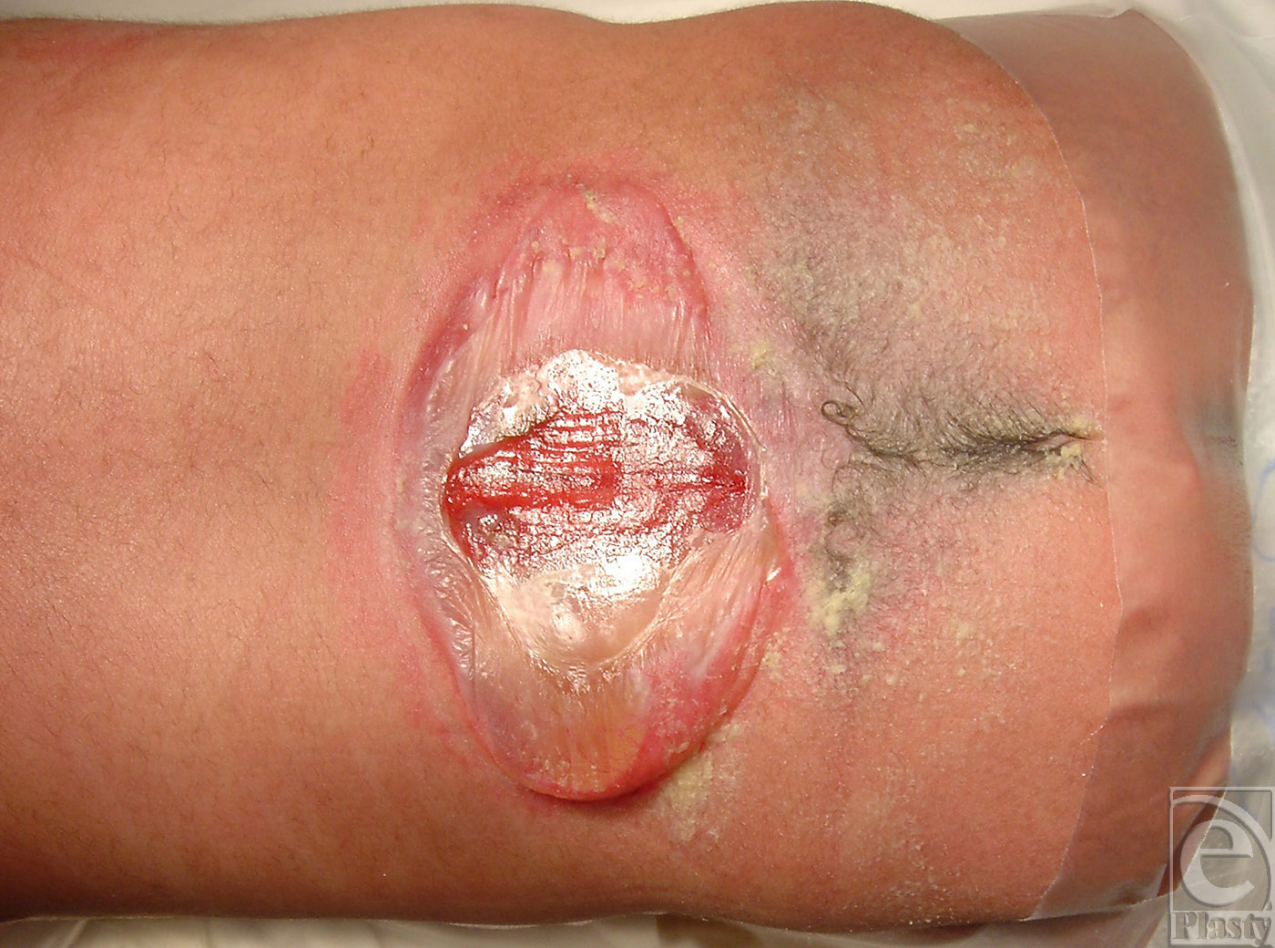
Large lumbosacral defect.

**Figure 6:**
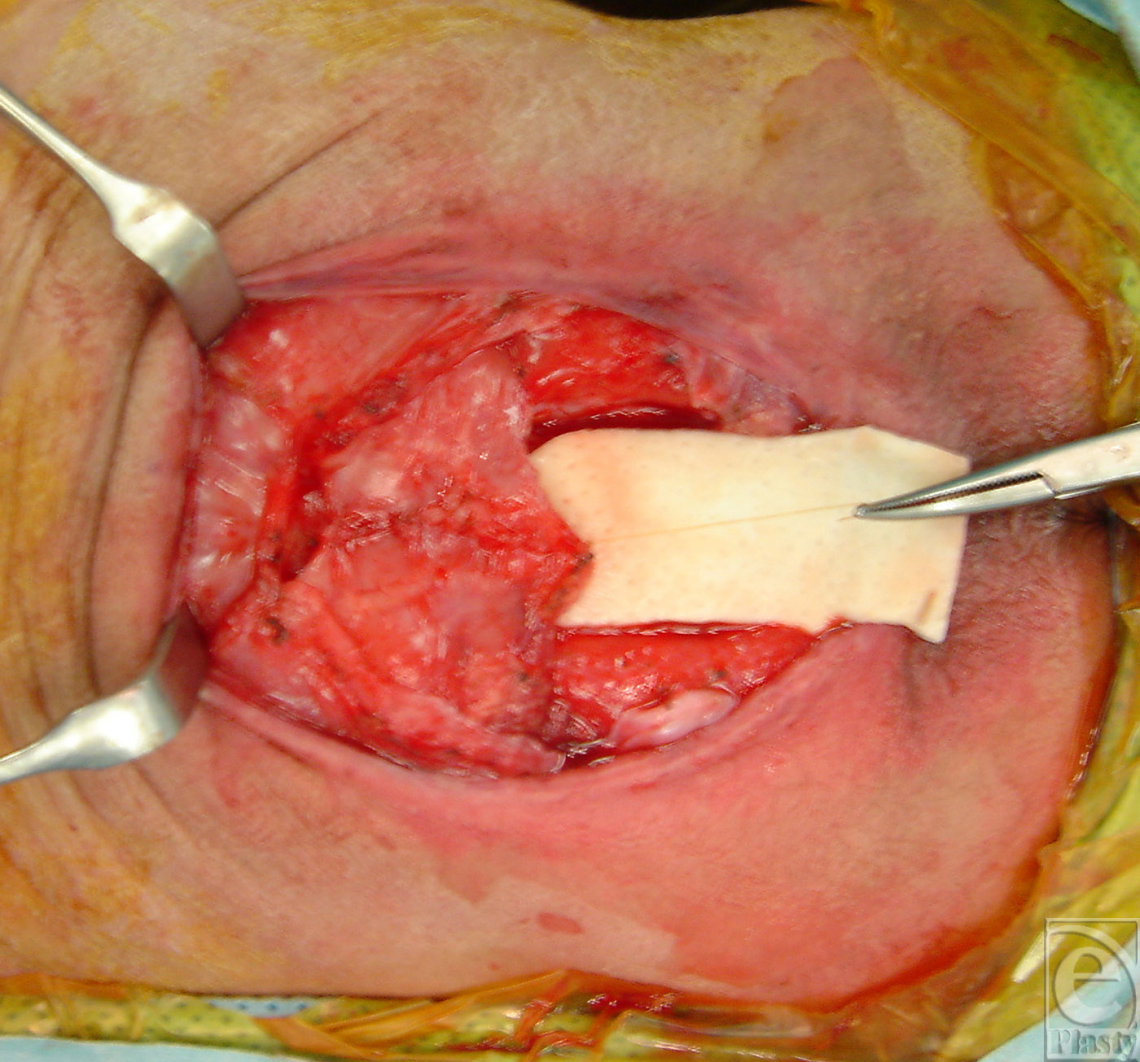
Placement of ACDM over dural repair, deep to fascial flap closure.

**Figure 7:**
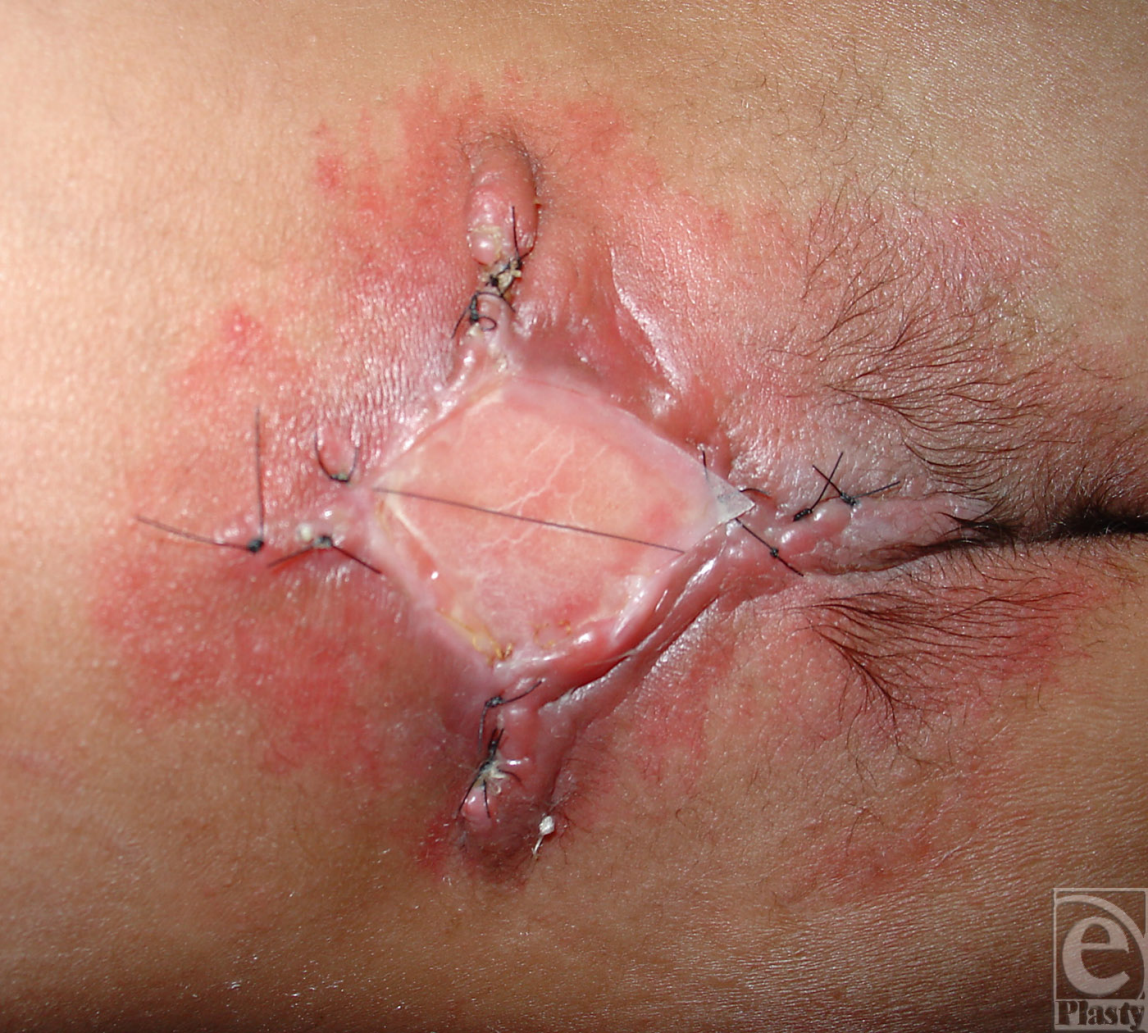
Placement of Integra R.

**Figure 8:**
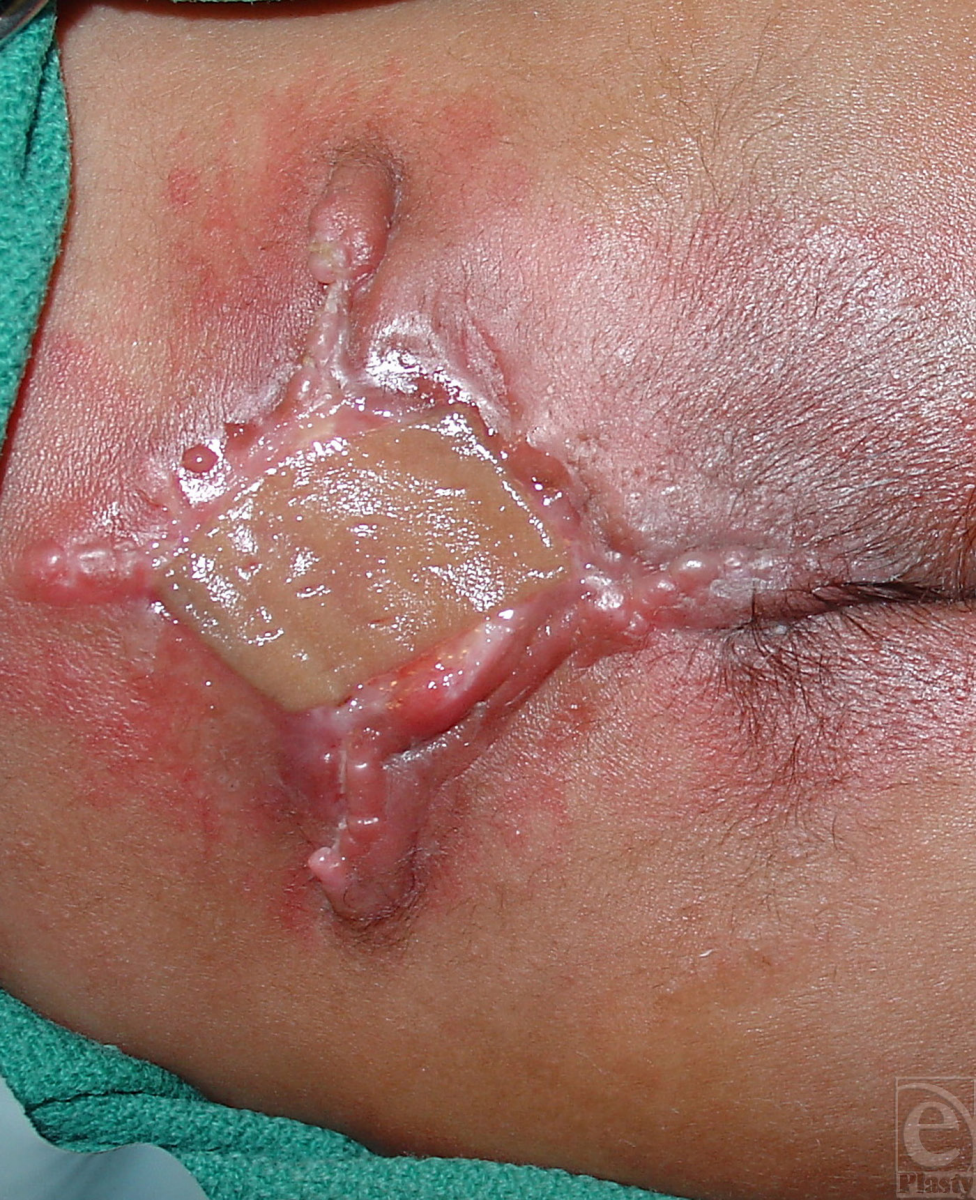
Placement of STSG.

This neonate was born at 37 weeks of gestation with a large meningomyelocele (Fig 5) and an associated cerebrospinal fluid (CSF) leak. The patient was brought to the operating room, and the dural defect was repaired by neurosurgery. After the repair, the wound measured 8 cm × 8 cm and the skin edges were thin and friable. Lumbar fascial flaps as well as latissimus dorsi flaps were raised. A 3 cm × 1.5 cm piece of ACDM was placed and anchored with 5–0 chromic catgut (Fig 6). This was covered with the lumbar fascia and the latissimus flap. The skin closure left a 3 cm × 2 cm central defect. This was fitted with a piece of Integra (Fig 7). The silicone backing was removed 4 weeks later and covered with a split thickness skin graft (Fig 8). The dura was covered by the ACDM throughout the patient's hospital course and at no time was exposed. The patient healed without complication.

## CASE REPORTS 4 TO 12

These 9 neonates were all born with large lumbosacral meningomyelocele defects greater than 5 cm in diameter. Pediatric neurosurgery repaired the neural tube defects and plastic surgery performed skin flap closures within the first 48 hours of life. A layer of ACDM was placed over the dural repair and under the soft tissue closure with the dermal-epidermal interface side of the ACDM facing toward the dural repair. All 7 patients healed without complications. The patients did not have any neurological complications or adverse reactions to the ACDM at 2- and 3-year follow-ups.

## DISCUSSION

Spina bifida is a common birth defect of the central nervous system with meningomyelocele seen in approximately 1 in 1000 live births.^2^ Ninety percent of the mortalities associated with spina bifida cystica are due to meningitis, hydrocephalus, and neurological complications.^3^,^4^ With smaller defects, neurosurgical correction and closure by a single team is sufficient. However, for larger defects, definitive operative management should be performed by a combined neurosurgical and plastic surgical team as to provide reconstruction and protection of the exposed spinal cord, elimination of any CSF leakage, and stable soft tissue coverage.^5^ The multiple layers should be dissected and closed separately. The techniques for neurosurgical repair of the dural defects have remained relatively unchanged for the past 20 years. Despite this, there still remain closure-associated nuances stemming from the variable size and configuration of the neural and overlying skin defects.^6^ Myriad reconstructive options are available to achieve skin closure after the neural elements have been closed appropriately. However, in 25% of cases that have defects too large to close linearly,^7^,^8^ providing adequate soft tissue cover while preventing wound breakdown and infection can present a challenge.^9^ A variety of flaps and skin graft procedures have been developed to address these issues. In the past, primary healing has been critical for the protection of neurological function and minimizing the risk of infection.^10^–^13^

However, in these cases where the defect is large or the skin surrounding the defect is tenuous, primary healing can be challenging, as wound dehiscence is common. Superficial wound dehiscence is the most common complication after a meningomyelocele repair.^14^ However, there are no studies in the literature that compare the wound complication rate to wound size. The wound should be followed closely for the leakage of CSF. The incidence of wound infections ranges from 1% to 1.5% and usually occurs 5 to 7 days after repair.^14^ Infants are also at risk for enteric bacterial infection due to fecal contamination leading to meningitis with sepsis. Therefore, it is paramount to ensure that if a wound dehiscence does occur, there is an extra layer of protection to prevent exposure of the neural elements. The ideal biomaterial to prevent dural exposure as well as reinforce the underlying neural tube repair should be similar to the surrounding tissue's physical qualities and biocompatible.

ACDM is processed from human cadaver dermis. This material has been used for reconstruction of dural defects during craniotomies when linear closure is not possible or harvesting from an autologous donor site is not feasible.^15^ The cadaveric skin obtained from tissue banks is thoroughly screened for human pathogens. The processing involves removal of the epidermis, cellular material, and histocompatability class II antigens without altering the biochemistry and architecture of the dermal matrix. Prion diseases are not a transmission risk because ACDM is harvested from non-neurological cadaveric tissues. The cellular and antigenic elements have been removed from the matrix,^16^ and then become repopulated with the host's cells and revascularized.^15^ ACDM is an off-the-shelf product that is commercially available in freeze-dried sheets that must be rehydrated immediately before use. ACDM has 2 distinct surfaces: the dermal-epidermal junction and the dermal-subcutaneous interface. The dermal-epidermal junction maintains a basement membrane and is thought to potentially prevent adhesion. Consequently, the dermal-epidermal junction is always placed against the dural layer to minimize the potential for tethered cord in the event that the dural closure breaks down the underlying spinal elements that are exposed. ACDM has also shown to decrease contracture during healing^17^,^18^ which could also potentially reduce the risk of cord tethering. Heterotopic calcification within the graft is not encountered because of the absence of cross-linking.^16^ An additional important feature of ACDM is that it does not require an immediate blood supply but can nourish overlying tissues by transmitting vital interstitial fluids. This was clearly demonstrated in case 3 in which Integra integrated directly into the ACDM before skin grafting.

Myriad plastic surgical techniques have been developed for repair of large meningomyeloceles including wide undermining with skin advancement, musculocutaneous flaps with split skin grafting of the donor defect, bilateral sliding muscle flaps, paraspinous osteomuscular flaps, and split thickness skin grafts.^19^ The decision to use a specific technique is dependent on the size of the defect, vascular supply, the ability to preserve the function of the underlying structures, as well as the long-term durability of the repair.^20^ However, all carry the risk of complications including wound breakdown and flap necrosis. This could lead to infection or exposure of the underlying dural repair with subsequent neurological sequelae. Desiccation is yet another potential sequelae of cord exposure that could lead to neurological deterioration.^9^ When used to cover the dural repair underneath the soft tissue closure, ACDM adds a layer of protection that could prevent exposure of the dural repair and potential CSF contamination in the event of a wound breakdown. A novel technique by Fiala et al 5 using lumbar periosteal turnover flaps showed the same benefits as with the use of ACDM; however, the periosteal flaps require careful patient selection dependent on sufficient lumbar vertebral periosteum. This is not the case with ACDM, which can be used in any patient and in combination with any soft tissue closure. As with the lumbar periosteal turnover flaps, ACDM can be expected to prevent CSF leakage and a subsequent pseudomeningocele. The added layer of protection could serve as reinforcement in the event of an inadequate dural closure as performed in case 1.

In our first reported case in 2004,^1^ ACDM and a split thickness skin graft were used to protect the dural repair after the original skin flaps dehisced. However, although there was an unanticipated loss of the split thickness skin graft, ACDM alone proved to be a sufficient protective barrier to cover the dura to allow for uncomplicated secondary healing. In the first and second cases reported here, exposed ACDM overlying the dural repair healed secondarily without complication. The third case involved using a piece of Integra to close the wound with ACDM underneath. Throughout the hospital course and during coverage of the Integra with a split thickness skin graft, the dura was covered with ACDM and never exposed. In the remaining cases, the overlying skin flaps/soft tissue coverage healed uneventfully. However, if they had failed to heal or if there was a wound dehiscence or necrosis of the skin flaps, there would have been an added layer of protection that could have prevented exposure of the dural repair and potential CSF contamination. We recommend the use of ACDM in combination with any of the various soft tissue closure techniques to add a layer of protection during repair of large meningomyelocele defects.
